# Ovarian carcinoma patient derived xenografts reproduce their tumor of origin and preserve an oligoclonal structure

**DOI:** 10.18632/oncotarget.5069

**Published:** 2015-07-31

**Authors:** Pierre-Emmanuel Colombo, Stanislas du Manoir, Béatrice Orsetti, Rui Bras-Gonçalves, Mario B. Lambros, Alan MacKay, Tien-Tuan Nguyen, Florence Boissiére, Didier Pourquier, Frédéric Bibeau, Jorge S. Reis-Filho, Charles Theillet

**Affiliations:** ^1^ Department of Surgical Oncology, Institut de Cancérologie de Montpellier, Montpellier, France; ^2^ Institut de Recherche en Cancérologie de Montpellier, Université Montpellier, Montpellier, France; ^3^ INSERM U1194, Montpellier, France; ^4^ Institut de Cancérologie de Montpellier, Montpellier, France; ^5^ Breakthrough Breast Cancer Research Centre, Institute of Cancer Research, London, UK; ^6^ Unité de Recherche Translationnelle, Institut de Cancérologie de Montpellier, Montpellier, France; ^7^ Department of Pathology, Institut de Cancérologie de Montpellier, Montpellier, France; ^8^ Department of Pathology, Memorial Sloan Kettering Cancer Center, New York, NY, USA

**Keywords:** ovarian cancer, PDX, CNC, mutations, oligoclonality

## Abstract

Advanced Epithelial Ovarian Cancer (EOC) patients frequently relapse by 24 months and develop resistant disease. Research on EOC therapies relies on cancer cell lines established decades ago making Patient Derived Xenografts (PDX) attractive models, because they are faithful representations of the original tumor. We established 35 ovarian cancer PDXs resulting from the original graft of 77 EOC samples onto immuno-compromised mice. PDXs covered the diversity of EOC histotypes and graft take was correlated with early patient death. Fourteen PDXs were characterized at the genetic and histological levels. PDXs reproduced phenotypic features of the ovarian tumors of origin and conserved the principal characteristics of the original copy number change (CNC) profiles over several passages. However, CNC fluctuations in specific subregions comparing the original tumor and the PDXs indicated the oligoclonal nature of the original tumors. Detailed analysis by CGH, FISH and exome sequencing of one case, for which several tumor nodules were sampled and grafted, revealed that PDXs globally maintained an oligoclonal structure. No overgrowth of a particular subclone present in the original tumor was observed in the PDXs. This suggested that xenotransplantation of ovarian tumors and growth as PDX preserved at least in part the clonal diversity of the original tumor. We believe our data reinforce the potential of PDX as exquisite tools in pre-clinical assays.

## INTRODUCTION

Epithelial Ovarian Cancer (EOC) is the leading cause of gynaecologic cancer-related mortality in women worldwide [1]. Its insidious progression explains why 75% of the patients present at diagnosis with tumour spread throughout the abdominal cavity [1]. Despite frequent complete clinical response, nearly all patients with advanced stages relapse after a mean period of 18 months and develop treatment resistant disease [1–3]. The prognosis of advanced EOC thus remains grim, with about 30% 5-year overall survival [1, 4].

EOC form a heterogeneous group of tumors. Classically ovarian tumors are stratified according to 5 histological types, tumor grading and disease stages. A recent classification defined two types of EOC; type I cancers largely including low grade and EOCs of rare subtype and type II corresponding to high-grade EOC, mainly of the Serous histotype [5]. Noticeably, whilst type I EOCs present frequent KRAS activation, occasional *TP53* mutations and a relatively favorable outcome, type II present a reverse picture with rare *KRAS* activation, over 95% of *TP53* mutations and an adverse outcome [6].

It is commonly observed that EOCs with identical histological type and tumor grades display different clinical courses and responses to treatment. The best documented differences are found between Serous Ovarian Carcinomas bearing a germline *BRCA1* mutation, which in most cases show increased sensitivity to platinum-based chemotherapy, and sporadic SOC devoid of *BRCA1* mutations, which respond poorly [7, 8]. Exceptions to this rule, however, are commonly encountered [8].

Additional biomarkers and novel treatment strategies are, therefore, urgently needed. To this end, reliable biological models are indispensable tools. At present experimental work on ovarian cancer relies on a limited set of cell lines, most of which established decades ago and, thus, susceptible to drift because of selection caused by culture conditions. Noticeably, xenografted cell lines form undifferentiated tumors that have lost the architectural organisation prevailing in the tumor of origin [9]. Furthermore, the recent work by Domcke and coworkers determined that a substantial fraction of the 47 ovarian cancer cell lines analyzed displayed genetic features distinct from those of the EOCs analyzed by TCGA [10]. All these elements call for novel ovarian cancer models to support experimental and preclinical work. In the last decade patient derived xenografts (PDX) have gained considerable interest. Seminal works in the field have demonstrated that PDX faithfully reproduced the histology and morphology of the tumor they stemmed from [11, 12]. We, and others, have since shown that PDX not only show excellent conservation of morphologic features, they also conserve their molecular characteristics [13–15]. These data added to the work by Hidalgo and coauthors showing that PDX perfectly mimicked response of the disease in the patient to a variety of chemotherapeutic drugs are strongly in favor of the generation of EOC PDX [16].

In this work we report the establishment of a collection of 35 ovarian cancer PDXs resulting from the original graft of 77 ovarian tumor samples onto immuno-compromised mice. Established PDXs covered the diversity of EOC histotypes. Genetic (array-CGH and transcriptome) and histological characterization of a subset of 14 PDXs revealed that PDXs perfectly reproduce the original genetic and morphological features of the ovarian tumors they stemmed from. PDXs conserved the principal characteristics of the original CNC profiles over several passages indicating genetic stability. Fluctuation of CNC pattern in a fraction of chromosomal regions that were observed between the original tumor and PDXs revealed that PDX globally maintained an oligoclonal structure. This could be confirmed by exome sequencing.

## RESULTS

### Ovarian carcinomas grafted and resulting PDX collection

We grafted a total of 77 fresh ovarian carcinoma specimens into the inter-scapular fat pad of Swiss-Nude mice at latest three hours after surgical resection. The 77 EOC samples grafted in this study were collected from 55 patients. Twenty-nine (29) tumor samples corresponded to multiple specimens collected from 10 patients at surgical debulking in different locations in the peritoneal tumor mass. Furthermore, 7/77 tumor samples collected from 5 patients corresponded to recurrences of a primary EOC previously engrafted (Table 1 and Supplementary Table 1 for full details). We assessed PDX take at passage 1 (P1) and at passage 3 (P3). Eleven and 16 grafts were under assessment (UA) at P1 and P3 respectively and were not included in the statistical analyses. We observed take at P1 in 47/66 (74%) going down to 35/61 (60.9%) at P3. This is consistent with previous observations with breast cancer PDXs, where graft losses in the P1 to P3 interval were common [13].

**Table 1:** Description of the EOCs grafted and their engraftment rate

	Patients	Samples grafted	Take P1	No Take P1	Take P1 vs No Take P1	UA P1	Take P3	No Take P3	Take P3 vs No Take P3	UA P3
	N	N	N (%)	N (%)	p value		N (%)	N%	p value	
Primary	55	55	37 (74%)	13 (26%)	N.S.	5	28 (60.9%)	18 (39.1%)	N.S.	9
Multilocation at first debulking	10	29	8 (66.7%)	4 (33.3%)		3	5 (45.5%)	6 (54.5%)		4
Recurrence	5	7	2 (50%)	2 (50%)		3	2 (50%)	2 (50%)		3
serous	45	67	38 (67.9%)	18 (32.1%)	N.S.	11	28 (53.8%)	24 (46.2%)	N.S.	15
Mucinous	2	2	2 (100%)	0		0	1 (50%)	1 (50%)		0
Clear cell	2	2	2 (100%)	0		0	1 (100%)	0		1
Carcinosarcoma	4	4	3 (75%)	1 (25%)		0	3 (75%)	1 (25%)		0
endometroid + serous	1	1	1 (100%)	0		0	1 (100%)	0		0
clear cell + serous	1	1	1 (100%)	0		0	1 (100%)	0		0
Grade 1	5	5	3 (60%)	2 (40%)	N.S.	0	1 (20%)	4 (80%)	N.S.	0
Grade 2	8	10	8 (80%)	2 (20%)		0	8 (80%)	2 (20%)		0
Grade 3	36	56	31 (68.9%)	14 (31.1%)		11	23 (56.1%)	18 (43.9%)		15
Grade ND	6	6	5 (83.3%)	1 (16.7%)		0	3 (60%)	2 (40%)		1
Stage I	4	4	3 (75%)	1 (25%)	N.S.	0	2 (50%)	2 (50%)	N.S.	0
Stage II	2	2	0	2 (100%)		0	0	2 (100%)		0
Stage III	45	67	40 (71.4%)	16 (28.6%)		11	31 (59.6%)	21 (40.4%)		15
Stage IV	4	4	4 (100%)	0		0	2 (66.7%)	1 (33.3%)		1
N-	14	16	9 (60%)	6 (40%)	N.S.	2	5 (35.7%)	9 (64.3%)	N.S.	3
N+	34	52	33 (73.3%)	12 (26.7%)		9	25 (61%)	16 (39%)		13
median age	62		64	61.5	N.S.	64	64	62	N.S.	65
age range	28-85		38-85	28-85		68-57	40.5-85	28-85		57-72
Recurrence in High Grade EOC										
< 6 months			5 (100%)	0 (0%)	0.008		5 (100%)	0 (0%)	0.02	
6 to 18 months			19 (86.4%)	3 (13.6%)			15 (68.2%)	7 (31.8%)		
>= 18 months			8 (47.1%)	9 (52.9%)			8 (47.1%)	9 (52.9%)		
Total	55	77	47 (71.2%)	19 (28.8%)		11	35	26 (42.6%)		16

EOC tumor samples were stratified according to the principal pathological and clinical characteristics of the disease. Take and No Take designates positive engrafment and tumor growth at passage 1 (P1) and passage 3 (P3) respectively. UA (under assessment) designates tumors grafted on animals and whose status is still pending. Grade ND not determined. Cases included in the recurrence analysis corresponded to clinically documented recurrence before 18 months or cases with no recurrence with a follow up of at least 18 months

The PDX models generated in this study covered the diversity of EOC, as all histological types except endometrioid carcinoma were represented by at least one specimen at P3. We also counted 2 PDXs with mixed histology presenting both histological components (Table 1, Supplementary Figure 2). Remarkably, 3/5 (60%) Grade 1 EOC showed take at P1, however, only 1 made it through passage 3. *TP53* mutation status was determined in a subset of 15 EOCs (Supplementary Table 1) and revealed missense mutations in 12/15 cases analyzed. The 3 *TP53* wild type cases were of the mucinous, clear cell and serous carcinoma type. These results were consistent with the high prevalence of *TP53* mutations reported in the TCGA EOC set [17].

We investigated whether positive engraftment and PDX establishment was significantly enriched in specific subsets of EOCs and noticed that neither histological type, tumor grade, FIGO stage nor nodal involvement showed statistical association with take at P1 or P3 (Table 1). Remarkably, however, engraftment appeared associated to disease aggressiveness, as early (< 6 months) and median term recurring EOCs showed a significantly higher take rate at P1 and P3 than late recurring (> 18 months) tumors. This correlation was reinforced in high-grade EOCs (Table 1). Kaplan-Meier analyses further strengthened the notion that EOC graft take was associated with negative outcome of the disease (Supplementary Figure 1A). Interestingly, PDXs that grew most rapidly after initial engraftment stemmed from EOCs with shorter overall survival (Supplementary Figure 1B).

### Conservation of the phenotypic features of the tumor of origin in EOC PDXs

**Figure 1:**
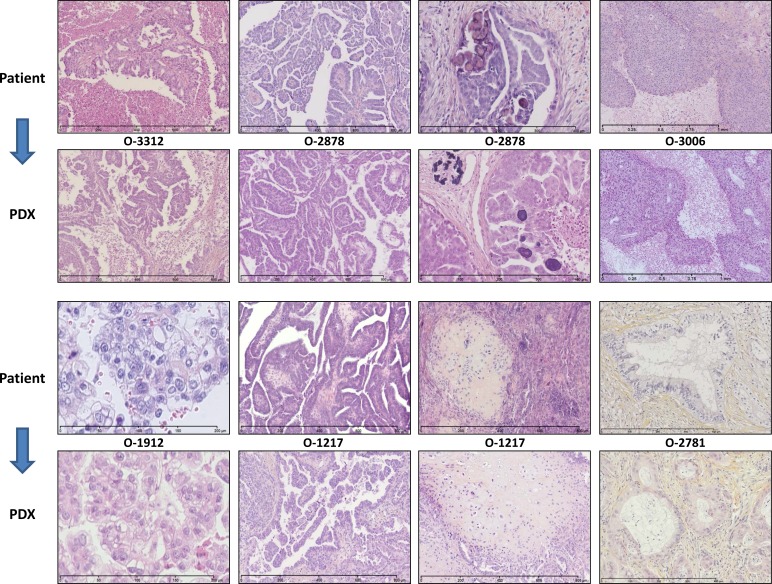
PDX faithfully replicate the morphology of the EOC of origin Most representative examples are shown here. O-3312 is a Grade 3 (Silverberg classification) Serous Carcinoma characterized by papillae proliferation and extended necrotic areas; O-2878 is a Grade 2 Serous Carcinoma with papillae architecture and psammoma; O-3006 is an undifferentiated Grade 3 Serous Carcinoma with solid architecture and large necrotic areas; O-1912 is a Clear Cell Carcinoma; O-1217 Grade 2 is a Carcino-Sarcoma (Mixed Mixed Müuuml;llerian tumors) showing inclusions of sarcomatous tissue within an undifferentiated epithelial carcinoma; O-2781 is a mucinous carcinoma with mucus material accumulating inside and outside tumor cells. Note that the characteristics observed in the original tumors were strikingly reproduced in the PDXs that were generated.

We characterized a subset of 14 EOC PDX models at the histological and molecular levels. Histological assessment by two pathologists with interest in gynaecological oncology, FB and DP, confirmed that the PDXs presented a remarkable conservation of the morphological features and differentiation level of the tumor they originated from. Our PDX panel is representative of the different histological types of EOC with a majority of high-grade SOC. Rare histological types are also represented with 1 clear-cell, 3 carcinosarcomas (Mixed Malignant Müuuml;llerian Tumors), and 1 mucinous tumor. Global architecture, histological type and grade of the tumor of origin were well conserved in the PDXs, which reproduced specific features such as papillae proliferation, necrotic areas or psammoma bodies (Figure 1, Supplementary Figure 2 for the complete set of 14 EOCs and cognate PDXs). PDXs showed variable proportion of stroma formed of normal murine cells, as demonstrated by FISH with human and murine-specific Cot-1 DNA hybridization probes (Supplementary Figure 3). Interestingly, PDXs recapitulated the intratumoral heterogeneity of the primary cancer they stemmed from. Most of the sampled serous carcinomas showed an admixture of grade 2 and grade 3 areas. This heterogeneity was observed in both the primary ovarian tumors and the peritoneal deposits, and was reproduced in corresponding PDXs at different passages (Figure 1 and Supplementary Figure 2). PDXs established from carcinosarcomas were another striking example of the faithful conservation of the histological structure of the patient’s tumor. Indeed, carcinosarcomas are characterized by the presence of sarcomatous areas within larger carcinomatous sections. Eventually, at relapse the sarcomatous contingent will take over. It is therefore of great interest to note that PDX stemming from carcinosarcoma reproduced the dual architecture of the original tumor (Figure 1 and Supplementary Figure 2).

Conservation of the original phenotypic characteristics in the PDXs was further confirmed by a transcriptome analysis performed on a subset of 9 EOCs and 11 corresponding PDXs. Expression data were analyzed by hierachical clustering and PDXs systematically co-clustered with their tumor of origin (Supplementary Figure 4).

### PDXs conserve the genomic profiles of the EOC of origin

**Figure 2:**
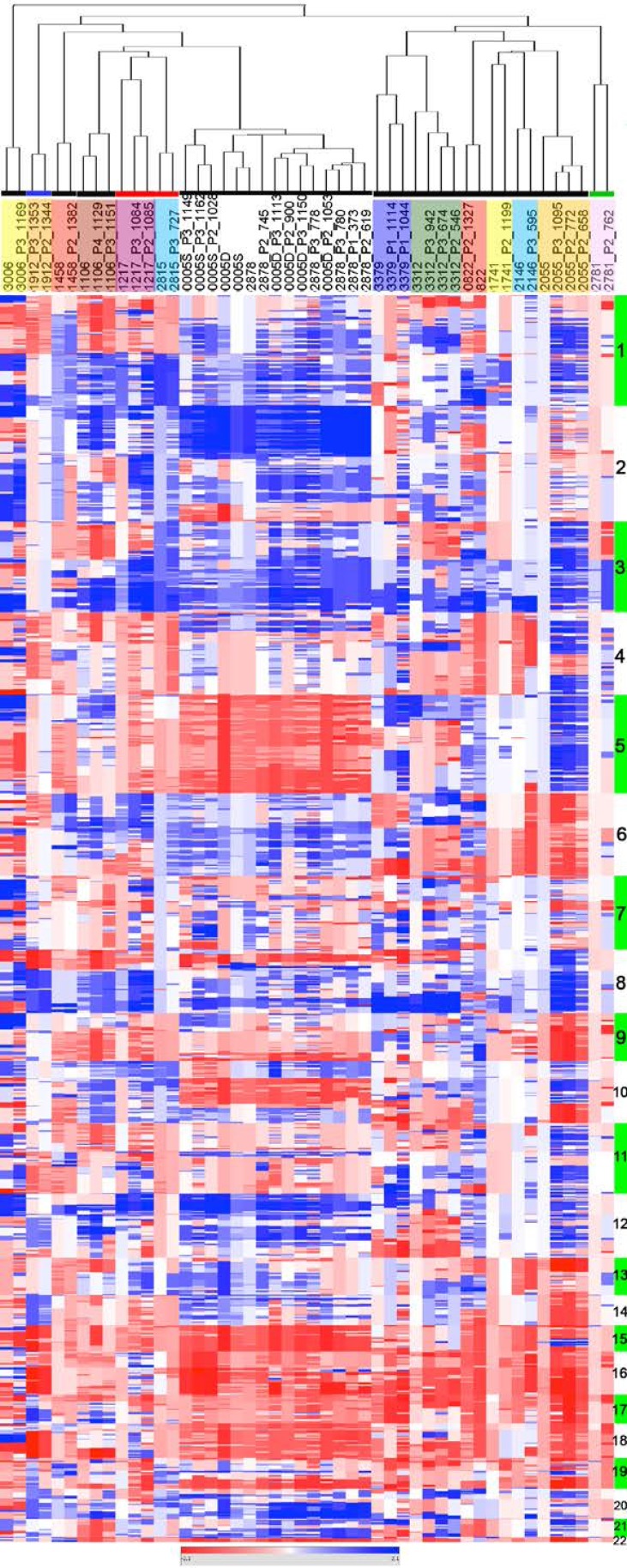
EOC of origin and corresponding PDXs show similar CNC profiles CNC profiles of patient tumors and corresponding PDXs were analyzed by unsupervised hierarchical clustering showing systematic co-clustering. When several PDXs generated from the same original tumor could be analyzed they regrouped as tight clusters. Samples are in columns, chromosomal localization in rows. Chromosomes are indicated as alternating green and white vertical bars and identified by their number. Each family of EOC and derived PDXs are highlighted in colored boxes below the dendogram (no code associated to the colors used). Colored horizontal lines indicate the histological type of the EOC grafted; black: serous ovarian carcinoma, blue: clear cell carcinoma, red: carcino-sarcoma, green; mucinous carcinoma.

Copy number change (CNC) profiles of 16 patient tumors and 33 PDXs resulting of their engraftment were determined by array-CGH on 32K tiling path BAC-array. CNC profiles of primary tumors and corresponding PDXs showed parallel evolutions reflected by similar profiles of aberrations that systematically co-clustered in a hierarchical clustering tree (Figure 2). Most prevalent CNC in our cohort were in concordance with ovarian cancer genomic profiles defined in the TCGA dataset [17]. Regions of high level gain or amplification encompassed key cancer genes such as *MYCN* (2p), the *EVI1*/*MDS1* cluster (3q), *FGFR1* (8p), *OCT4* and *MYC* (8q) and *KRAS* (12p) of which a number are potential therapeutic targets. Despite the similarity revealed by the clustering analysis, a close examination of the profiles (Heatmap in Figure 2 and Supplementary Figure 5) revealed focal differences between the PDXs and their tumor of origin. A global increase of the CNC number in the PDXs (median = 156, range 47-460) compared to patient tumors (122, range 29-297) was noticeable. This could in great part be explained by sizeable fractions of contaminating normal cells in some of the patient samples, which affected the correlation coefficient between the tumor of origin and corresponding PDXs (Supplementary Figure 6). This was also associated to an increase of the amplitude of gains in the PDXs (Heatmap in Figure 2). However, a number of patient tumors presented CNCs that were not scored in the PDX, suggesting oligoclonality of the tumors of origin (Figure 2 and Supplementary Figure 5, sample 1912, 1458, 3312, 2878). We thus decided to investigate this aspect in further detail.

### PDX revealed substantial levels of oligoclonality in EOCs

**Figure 3:**
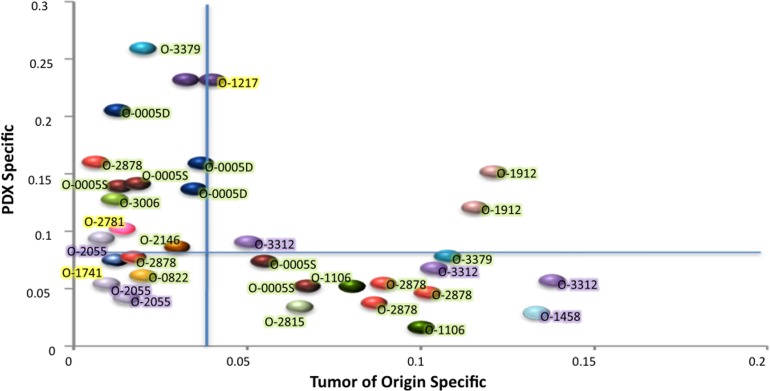
Fraction of the genome involved in CNCs differing in the PDX as compared with cognate patient EOC The X axis represents CNC originally present in the patient tumor and absent in the PDX (Tumor of origin specific), the Y axis CNCs occurring de novo in the PDX while not scored in the tumor of origin (PDX specific). Vertical and horizontal blue lines indicate the median levels of tumor specific (X) or PDX specific (Y) CNCs. Each color ball represents the comparison of the tumor of origin and one PDX. Tumors occurring only once in the graph are those where only one PDX was analyzed. Tumor IDs are indicated and color highlights indicate the fraction of tumor cells for each tumor of origin; green 70 to 90%, purple 50 to 70%, yellow < 50%.

The fraction of the genome involved in CNCs differing in each PDX and in the tumor of origin was determined and stratified as (i) events occurring *de novo* in the PDX (PDX specific) and (ii) events present in the original tumor and lost in the PDX (tumor specific) (Figure 3, Supplementary Figure 7). According to the PDX analyzed, *de novo* events represented 3% to 26% of the genome, whereas loss of tumor specific CNCs ranged from 2% to 13% of the genome. Overall these data indicated the genetic plasticity of PDXs established from EOC. Interestingly, most PDXs showed either a prevalence of de novo events (Y axis) or loss of tumor specific events (X axis), but rarely combined both (Figure 3). The second point of notice was that *de novo* CNCs were not linked to low levels of tumor cells in the tumor of origin. As a matter of fact, 9/11 PDXs showing more than 10% of the genome involved in *de novo* events stemmed from EOCs with 75 to 90% tumor cells, strongly suggesting that these events arose from the oligoclonal background present in the tumor of origin (Figure 3). These 9 cases, combined with the 14 PDXs showing loss of tumor specific events, strongly suggested that 10/16 (62.5%) of the grafted ovarian EOCs bore an oligoclonal structure.

### Genetic plasticity in ovarian cancer patient samples and derived PDXs

**Figure 4:**
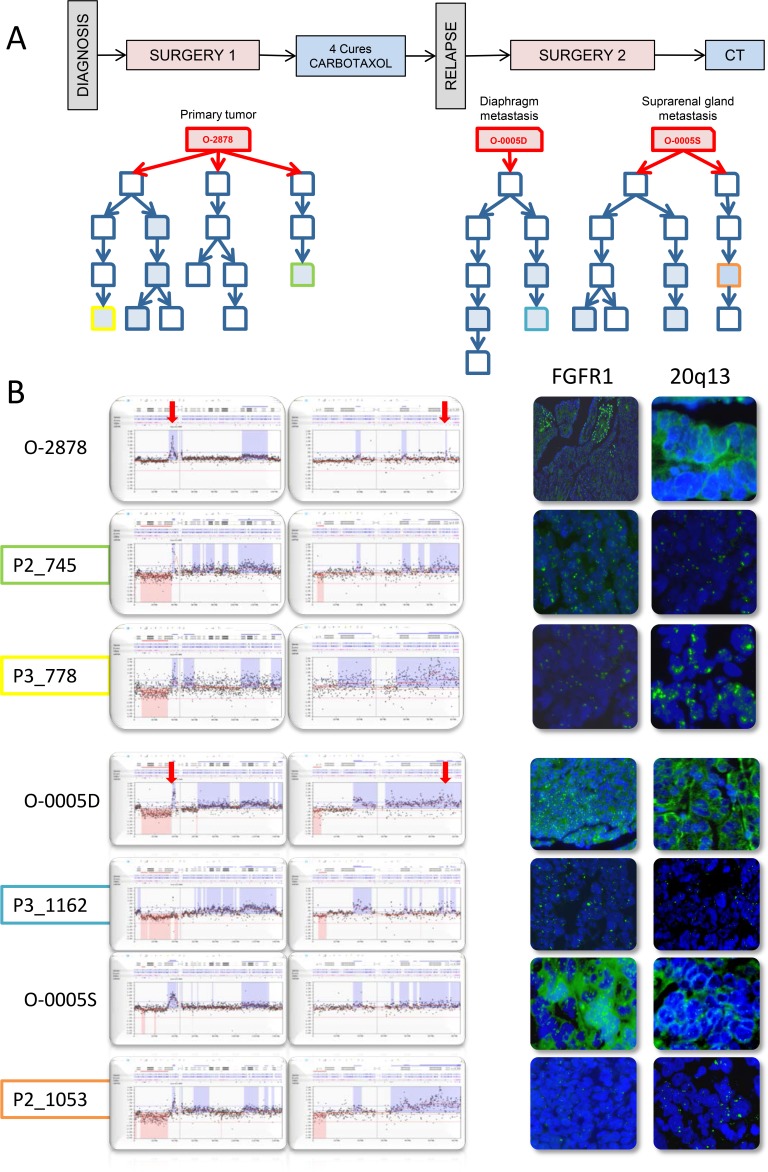
PDX established from tumor O-2878 and subsequent metastatic recurrences O-0005D and O-0005S show fluctuating CNC patterns at 8p12 and 20q13 indicating oligoclonality **A.** Schematic representation of the clinical history of patient 2878 and graft trees of the PDXs established from the 3 original tumors. Each represents a PDX. Boxes in blue filling correspond to cases that were analyzed by array-CGH, color lining identifies PDXs presented in B. **B.** zoomed representations of array-CGH profiles at chromosomes 8p12 (left box) and 20q13 (right box) showing variation in copy number levels as well as in the size of the region of gain. Variations observed by array-CGH were confirmed by interphase FISH on frozen tumor sections using probes to FGFR1 (8p12) and an anonymous probe to 20q13.

**Figure 5:**
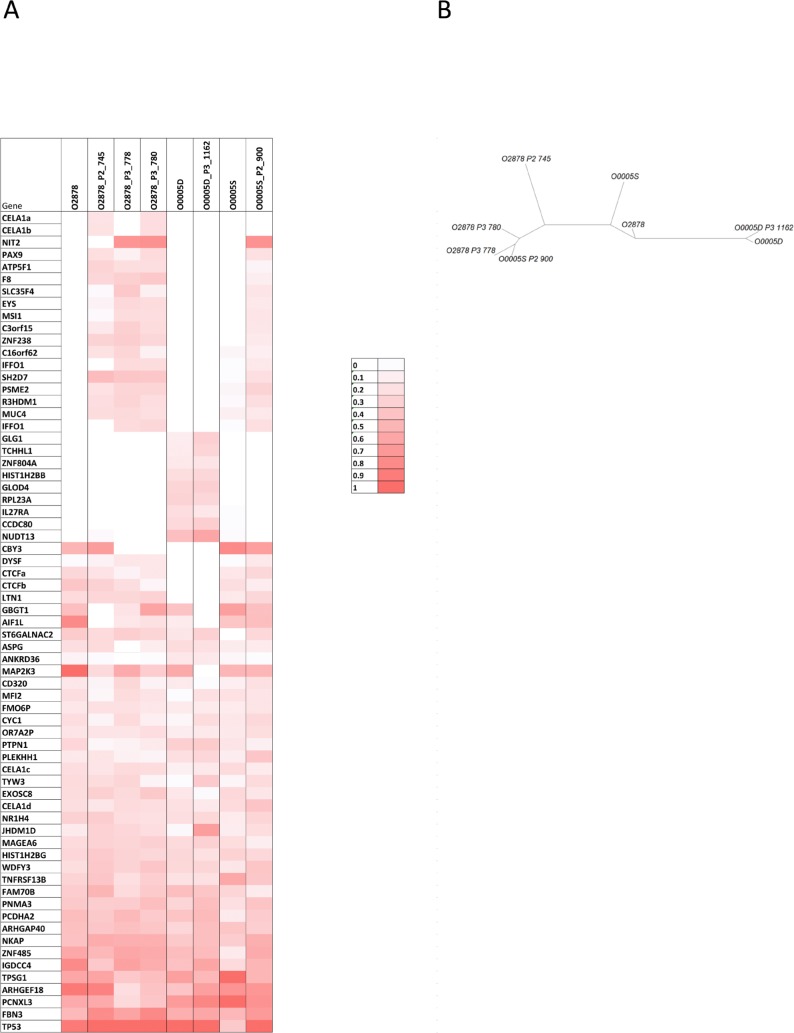
mutational profiles of O-2878, distant nodules O-0005D and O-0005S and the PDX that were derived from them **A.** mutations detected were indicated in shades of red ranging from dark red (100% of the calls) to light red (10% of the calls). White boxes indicate samples in which the mutation was not detected. **B.** Phylogenetic tree of the 3 tumor samples and their PDX offsprings. The 3 patient samples show greater proximity to each other than they do to their corresponding PDXs.

O-2878 and its associated distant tumor nodules O-0005D and O-0005S constituted interesting study cases of genetic evolution. O-2878 was a grade 2 serous ovarian carcinoma removed from a 64 year-old patient by primary surgical debulking prior to chemotherapy. Three animals were successfully engrafted at the time of primary surgery. After 4 cycles of chemotherapy, the patient underwent interval surgery at which two distant nodules were removed from the diaphragm (O-0005D) and the suprarenal gland (O-0005S). Both nodules were successfully engrafted on 1 and 2 animals respectively. Five PDX lines were produced and, upon serial passages, generated 9 parallel branches (Figure 4A and Supplementary Figure 8). Twelve PDXs were sampled from the different branches for CNC analysis and revealed significant genetic plasticity (Figure 3). Interestingly, nodules O-0005D and O-0005S showed greater proximity (illustrated by higher Pearson correlation coefficients) to the primary tumor O-2878 than did any of the PDXs to their tumors of origin (Supplementary Figure 8). Variations from one branch to another frequently concerned levels of gains and most representative changes were amplification at 8p12 and gains at 20q13 determined by array-CGH and confirmed by FISH analysis on tissue sections (Figure 4B). Moreover, we noted that changes in CNC levels were concomitant with remodeling of the size of the amplified region that could vary substantially from one sample to another (Supplementary Figure 9). To further assess clonal variations we performed exome sequencing at a mean 30X depth on the 3 patient tumors and a selection of 5 cognate PDXs. Normal blood DNA from this patient was used as germline reference. A total of 67 mutations corresponding to non-synonymous single nucleotide changes or Indels with an impact on the protein sequence were detected in 65 genes. Of the 67 mutations, 40 were found in the primary tumor, whereas 27 were not detected in the primary tumor and observed in at least 2 PDX and/or distant nodule (Figure 5A). Twenty nine (29) mutations were shared by all the samples thus corresponding to the ancestral tumor clone. Overall, these data substantiated the notion of clonal variation in these samples. Indeed, only the *TP53* mutation was highly enriched (mutant allele fraction of 92%) in the primary tumor and reached homogeneity (mutant allele fraction of 100%) in the PDXs, indicating a strong selective advantage. The mutant allele fractions of all other mutations fluctuated according to the sample tested (Figure 5A). For instance, mutations in the *IGDCC4*, *ARHGEF18*, *MAP2K3* or *AIF1L* genes, which were observed in fractions exceeding 80% in the primary tumor ranged from 0 to 86% in the PDXs or secondary nodules (Figure 5A). The 18 mutations that were not found in the primary tumor but observed in its PDX could be interpreted as mutations newly emerging in the PDX, however, it is noticeable that 16/18 mutations were also observed in a PDX derived from O-0005S. This is in favor of mutations present at a low prevalence in the primary tumor and whose prevalence increased in PDXs, possibly resulting from growth conditions. Phylogenetic analysis revealed that the primary tumor O-2878 shared the greatest proximity with nodule O-0005S and second with nodule O-0005D, while PDXs formed downstream branches (Figure 5B). These results indicate that although PDXs displayed clonal variations they conserved at least in part the genetic diversity originally present in the tumor they stemmed from.

## DISCUSSION

Patient derived xenografts (PDX) are considered to be closer representations of the disease than cancer cell lines or genetically modified mice, making them increasingly appreciated models in preclinical tests [18]. This has encouraged a number of laboratories to undertake PDX establishment, as reflected by a flourishing literature presenting model collections in breast [12, 13, 19–21], lung [22–25], pancreatic [26, 27], renal cancers [28], uveal melanoma [29] and gynaecologic tumours [30].

Epithelial ovarian carcinoma (EOC) is a devastating disease because of its silent progression. Most patients recur 18 to 24 months after chemotherapy and eventually develop resistant or refractory disease. Hence, there is a need to develop novel therapeutic approaches and representative models of this malignancy. To this end, we and others have taken up the challenge to create a panel of PDX models covering the genetic and phenotypic diversity of EOC [31–33]. Our collection, totalling 35 established PDXs, is to our knowledge the second largest collection of EOC models reported. As illustrated by the positive take of rare ovarian cancer subtypes such as clear cell, mucinous or carcinosarcomas, our PDX collection covered the diversity of EOC. We noted, however, the absence of endometrioid carcinoma in our collection, which may be related to the fact that early stage EOC patients tend to be referred to other institutions than ours. We registered an overall take rate of 60% at passage 3, which appeared lower than the 74 to 85% reported in other studies [31, 33]. One explanation to this observation may be that we used nude mice as recipient animals rather than SCID or NOD-SCID in other studies. Graft site has also been a point of discussion. Some groups favored intraperitoneal (IP) grafting, because of its proximity to the clinical situation and the possibility to monitor peritoneal spread [31]. We grafted EOC specimen into the inter-scapular fat pad as it allowed simple monitoring of graft take and tumor growth in the absence of ultrasound device. Data by Dobbin and coworkers [33] showing a better take rate upon subcutaneous *versus* intraperitoneal implantation suggest that our choice could have been pertinent.

Noticeably, we observed that EOCs from patients with early recurrence and death showed increased take rate. This was consistent with earlier data, on EOC [31], breast cancer [13] and lung cancer [25] indicating that clinically aggressive cancers have increased prospects of engraftment and grow faster on mice. This suggests the existence of biological determinants that favor both early recurrence in the patient and positive engraftment in the mouse. This may correspond to specific sets of genetic anomalies or the presence of larger contingents of tumor initiating cells in these tumors. Remarkably however, Dobbin and coauthors [33] did not observe any increase of ALDH1 and/or CD133 positive cells in the PDX in comparison to original tumors, concluding that PDX reproduced the cellular diversity that preexisted. This is of note because it suggests that graft take and subsequent passaging do not result in the selection of the most aggressive and undifferentiated cell subpopulation. Our work supports this, as we observed remarkable conservation of the architecture and cellular composition of the original tumor in the PDXs. Indeed, PDX recapitulated the phenotypic heterogeneity of tumors showing admixtures of cell contingents with different levels of differentiation or different phenotypes (areas of sarcomatous cells embedded in carcinoma) and did not select out the least differentiated subset. This is in agreement with the conservation of the phenotypic traits of the tumor of origin in the respective PDX models that was observed at both the histological and gene expression level.

In agreement with previous works by us and others [13, 15, 31], we observed in all PDXs tested an excellent reproduction of the genomic characteristics of the tumors they stemmed from. CNC profiles of the tumor of origin were recapitulated in the PDXs, showing only minor changes over passages. These changes corresponded to variations in copy numbers affecting specific subregions that we could clearly attribute to the oligoclonal structure of the tumors of origin. Overall, we estimated that oligoclonal CNCs represented up to 15% of the genome and affected 10/16 (62.5%) grafted patient samples that we analyzed at the genomic level. Levels of oligoclonality observed in this set of EOC were higher than those previously estimated in our breast cancer PDX study [13]. Interestingly, different PDXs derived from the same tumor of origin showed fluctuations in CNC and particularly in regions of gains suggesting that PDXs maintained an oligoclonal structure along passages. Exome sequencing data were in agreement with CNC observations, given that they indicated that different PDXs established from the same series of patient samples maintained the genetic diversity present in the tumor of origin. Remarkably, we did not observe any clear selection bias of mutations that would be preferentially found in the PDXs. We consider that this finding is important for the representativeness of PDXs as disease models. Indeed, recent studies of human cancer have pointed to tumor oligoclonality as a major hurdle to therapy efficacy [34, 35]. Ovarian cancer generally shows favorable initial response to chemotherapy, however followed by a high rate of recurrence. Recent work by Bashashati and coworkers, demonstrating important intratumoral clonal variations in EOC, are strongly in favor of the notion that oligoclonality should impact on ovarian cancer response to treatment [36]. Hence, the fact that PDXs maintain an oligoclonal structure similar to that of the tumor of origin make them models of choice to study the impact of therapeutic regimens. Data by Dobbin and coworkers showing an increase of the fraction of CD133 expressing cells in PDXs that had undergone treatment further support this idea [33].

Thus, our findings, along with those from previous works demonstrating that PDX models have similar phenotypic features to those of the tumor of origin and respond to chemotherapy in a similar manner [32, 33], are strong arguments in favor of these models as a platform for pre-clinical testing. However, in line with previous observations on EOC PDX [31, 33] we found that tumor stroma in PDX was exclusively composed of murine cells. This is in disagreement with reports suggesting that human stromal cells are maintained for several generations in pancreatic cancer PDX [37]. Whether the maintenance of human stroma could depend on the tumor type remains to be defined, however it appears than the absence of human stroma and particularly of immune or inflammatory infiltrate may be considered as a limitation, since it excludes the use of PDX models in testing immune based cancer therapy.

## MATERIALS AND METHODS

### Patients and ovarian tumours

From January 2009 to January 2014, 77 tumours samples from 55 patients receiving surgery for EOC in the ICM/Val d’Aurelle Cancer Center (Montpellier, France) were collected from the Pathology Department upon macroscopic dissection and transferred to the animal facility and implanted within a maximum of 3 hrs after surgical removal. This study was reviewed and approved by the ICM/Val d’Aurelle Institutional Review Board and informed consent was obtained from all patients. Samples were systematically anonymized. Median follow-up time was 21 months. Full description of the grafted tumor samples is provided in Supplementary Table 1.

### Establishment of ovarian cancer PDX

A fragment of tumor (~8 mm^3^) was implanted into the inter-scapular fat pads of 3-4-week-old female Swiss-nude mice. Tumors were passaged onto a further cohort of mice before graft volume reached 2,000 mm^3^. The study was reviewed and approved by the ethics committees of the IRCM and the University of Montpellier animal (CEEA-LR-12028). Grafting and serial passaging were done as previously described [13]

### Histological analysis

Histology was assessed on hematoxylin-eosin stained sections by FB and DP pathologist at the Cancer Center of Montpellier (ICM) with an interest in gynaecologic cancer.

### DNA and RNA extraction

DNA and RNA were isolated from frozen tissues using the QIAmp DNA Mini kit and Rneasy Mini Kit (Qiagen S.A. France, Courtaboeuf, France). Each DNA sample was quantified by nanospectrophotometry (NanoView, GE Healthcare, Orsay, France) and qualified by 0.8% agarose electrophoresis. Qualification of mRNA was performed using a Bioanalyser (Agilent, Santa Clara, CA, USA).

### Array-CGH

The 32 K BAC-array tiling path collection platform was constructed at the microarray laboratory of Breakthrough Breast Cancer Research Centre, as described previously based on the 32,000 BAC clone collection from Children’s Hospital Oakland Research Institute (CHORI) (http://bacpac.chori.org/) spaced at approximately 50 kb intervals throughout the genome [17]. DNA labelling, array hybridizations, and image acquisition were performed as previously described [18]. Array-CGH data were processed from GPR files using the Nexus 6.1 Software (Biodiscovery, El Segundo, CA, USA). Analysis settings for data normalization, segmentation and calling were the following: normalization by lowess (smoothing 0.1), significant threshold for FASTST2 Segmentation algorithm: 1.0E-5, Max Continuous Probe Spacing: 1000, Min number of probes per segment: 3, high level gain: 0.485, gain: 0.138, loss:-0.153, homozygous copy loss:-0.73.

### Hierarchical clustering analysis

Array-CGH data segmented log2 ratio data were exported from the Nexus software and individual segmented profiles were merged using Merge bedgraph files function from Galaxy resulting in a file compiling about 7600 segments comprised between copy number transitions (breakpoints) found in all samples. Agglomerative hierarchical clustering of samples was done using pairwise average linkage and Pearson correlation as metric with the HierarchicalClustering function of Genepattern (Broadinstitute) which is an implementation of Cluster (http://rana.lbl.gov/EisenSoftwareSource.html).

### Gene expression profiling

Gene expression profiling was performed using the Illumina’s Gene Expression Arrays HumanHT-12.v4 following the manufacturer’s protocol. Samples were normalised to 100ng and were processed according to the Illumina Whole-Genome Gene Expression Direct Hybridisation Assay Guide, using the Ambion Kit: Illuminaüreg; TotalPrep™-96 RNA Amplification Kit (Illumina Direct Hyb Gene Expression). Qualitative and quantitative quality controls were performed on the labelled cRNA (Nanodrop for quantification of RNA, Agilent 2100 bioanalyser: RNA 6000 pico assay) and 1.5ug of labelled cRNA was subsequently hybridised to Illumina HumanHT-12.v4 Beadchip and scanned by the BeadArray Reader. The array intensity data was loaded into the Illumina GenomeStudio software v2010.2 and then visualised and normalised. All analyses reported here use the ‘quantile’ normalisation method with background correction within GenomeStudio (Illumina expression array direct-hyb basic bioinformatics analysis). Full details on RNA amplification and hybridisation can be found at www.illumina.com.

Raw gene expression values were robust-spline normalised using the Bioconductor lumi package (http://www.bioconductor.org/packages/2.3/bioc/html/lumi.html) in R. Genes were mapped to their genomic location using the lumiHumanAllv2 annotation database available from Bioconductor. Only Illumina transcript probes with detection P values\0.01 in [25% of samples were included; this resulted in a dataset of 12,699 transcriptionally regulated probes with accurate and unequivocal mapping information.

### Fluorescence *in situ* hybridization (FISH)

Fluorescence in situ hybridization (FISH) for FGFR1 was done using the ready-to-use commercially available digoxigenin-labeled ZytoDotüreg; SPEC FGFR1 Probe (Zytovision, Bremerhaven, Germany). The 20q13 probe was constructed as described previously [19] and biotin-labeled. Pretreatment, digestion, and hybridizations were done as described by Lambros et al [19]. Cot-1 DNA FISH: Mouse specific Cot-1 DNA (Life Technologies, Saint-Aubin, France) and human specific Cot-1 DNA (Roche, Meylan, France) were labelled respectively by using FISHBRIGHT 550 (red) and FISHBRIGHT 495 (green) labelling kits (Leica, Nanterre, France). A mixture of 100 ng of each labelled Cot-1 DNA was denaturated and hybridized at 37üdeg;C overnight on pretreated and denatured tissue slides. Briefly, 4ümicro;m thickness tissue sections were deparaffinized and then treated using Tissue Digestion kit I according to provider recommendations (Leica, Nanterre, France). Pepsin was incubated at RT for 10 minutes. Hybridization was observed using Leica fluorescence microscope and images were captured using CCD camera drived by Isis software (Metasystems, Altlussheim, Germany).

### Exome sequencing analysis

Samples were sequenced on the HiSeq using the Illumina standard exomic sequencing protocol based upon the Agilent SureSelect 50Mb V4 probe capture set and passed through CASAVA QC. Paired end reads were aligned to the human genome (hg19) and the mouse genome (mm9) with BWA using largely default parameters - Illumina PHRED scores and allowing single gaps and small in-dels. To deal with possible contamination by mouse DNA, paired ends reads that mapped to the mouse genome were excluded from further analysis [20]. BAM files from BWA were then run through the GATK Best Practice Variant Calling pipeline (v2), recalibrating and realigning around SNPs and Indels using dbSNP130. Recalibration was run either for all samples or pairwise for each tumour against the germline. Variants in both normal and tumor genomes were recorded in variant Call Format (VCF) and annotated with the Variant Effect Predictor script (ensembl).

Variants were filtered for those within the exon capture probe set (SureSelect_All_Exon_50mb_with_annotation.hg19) and somatic events were identified by removing those with a homozygous reference genotype in the germline sample. Variants were further filtered for GATK “Exome” false positives and annotated for depth and rate of each allele in paired genomes (T/N). Coding variants were selected as those altering the protein coding sequence (NON_SYNONYMOUS_CODING, STOP_GAINE, STOP_LOST and FRAMESHIFT_CODING). Multiple consequences were concatenated and somatic mutations counted at a variant level and a gene level in all of the samples. Variant which were at least covered by 4 normal reads and 4 variant were conserved. Supplementary Table 2 presents the variants which are present at least in two samples. For all the variants which appear missing in one sample, we checked manually using IGV and if two or more reads were found to support the variant we included the percentage of variants allele in the Table. Phylogenetic tree were produced similarly to [21]. Phylogenetic tree for mutation was generated from a binary matrix containing the mutations of all samples (rows) with ‘1’ and ‘0’ representing the presence and absence of a mutation in a gene (column), respectively. We built the tree using 1-pearson coefficient correlation as distance, and the Neighbor-Joining method of Saitou and Nei [22] and the Unweighted Pair Group Method with Arithmetic Mean (UPGMA) method of clustering tree. Construction and plotting the phylogenetic trees were done in R with the “ape” package [23].

### TP53 and KRAS mutation analysis

Mutations in exons 4, 5, 6, 7, 8 and 9 of *TP53* and in codon 12, 13 and 63 of *KRAS* were detected by Sanger sequencing. Primers for TP53 were as described [24] and listed in Supplementary Table. The primers for *KRAS* are listed in Supplementary Table. Procedure was as described in 50 ng cDNA was amplified and sequencing reactions were carried out using the DNA Sequencing Kit BigDye Terminator v 1.1 Cycle Sequencing Ready Reaction Mix (Applied Biosystems, Warrington, UK), as previously described. Sequences were analysed with Mutation Surveyor software (Softgenetics, PA, USA). Mutations were confirmed by repeat PCR and sequencing of forward and reverse strands.

## SUPPLEMENTARY MATERIAL FIGURES AND TABLES






