# Supplementary material for: Ovarian carcinoma patient derived xenografts reproduce their tumor of origin and preserve an oligoclonal structure
Source: Oncotarget. 2015 Jul 31;6(29):28327–40. doi: 10.18632/oncotarget.5069 (PMC4695063; doi:10.18632/oncotarget.5069)
Supplement: Supplementary file 1 [file oncotarget-06-28327-s001.pdf]

## Ovarian carcinoma patient derived xenografts reproduce their tumor of origin and preserve an oligoclonal structure

**Supplementary Material**

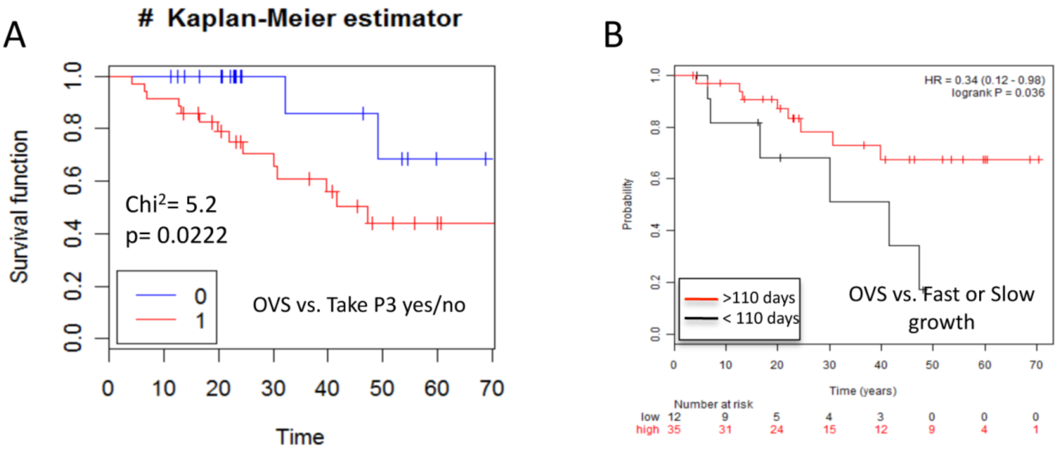


**Supplementary Figure 1**: Positive engraftment and rapid growth of the tumor on the animal are associated to shortened overall survival. A: patient tumors were stratified according to the capacity to establish a PDX model or not. Two groups, take and no-take, were defined and overall survival of the patients in either group analyzed. Survival of the take group was significantly shorter than those in the no-take group (Chi2 test p=0.022). B: Patient tumors were stratified according to the growth of the PDX that were generated from them. A fast and a slow growth group were defined (threshold 110 days between 2 passages) and overall survival of the patient compared. Survival of the patient in the fast growth group was found significantly shorter than that of the slow group growth group (logrank test p=0.036)

**Supplementary Figure 2:** PDXs faithfully recapitulate the histological architecture of original tumors.

**O-2055**

Poorly differentiated SOC (grade 3) containing moderately differentiated areas (25%) characterized by papillary and micro-papillary structures with psammoma bodies. Undifferentiated areas show packed tumor cells, nuclear atypias and vacuolar degeneration. The mean mitotic count is 10 mitoses/10 high-power fields (400x).

The PDXs recapitulate the morphology and the heterogeneity of the primary tumor with moderatly and poorly differentiated areas.

**O-2146**

High-grade SOC with papillary structures and diffuse/solid highly cellular growth pattern., marked nuclear atypias. Mitotic count is greater than 10 (400x).

**O-2815**

Mixed Müllerian tumor (MMT) or carcinosarcoma displaying epithelial portions with solid pattern, numerous mitoses and large necrotic areas. Papillary architecture is observed in the primary tumor. Within the epithelial component, chondrosarcomatous areas are identified. Poorly differentiated areas and the sarcomatous component predominate in tumor deposits sampled from the great omentum.

PDXs recapitulate the heterogeneous morphology with solid architecture and large necrotic areas, papillary morphologies as well as dispersed chondrosarcomatous areas.

**O-2878 and 0005D/0005S**

Moderately differentiated (grade 2) SOC containing 25% of poorly differentiated areas. Cellular atypias with high mitotic activity as well as psammoma bodies are present on peritoneal and pelvic deposits. Necrotic areas are absent on sampled sections.

Suprarenal and diaphragmatic post-CT nodules (O-0005S and O-0005D) sampled at interval surgery after chemotherapy conserve this moderately differentiated serous architecture, associated with solid areas and cellular atypias and intense mitotic activity.

PDX derived from the primary tumour reproduce the serous papillary and micro-papillary architecture and necrotic nucleus. PDXs derived from 0005S and 0005D bear comparable papillary architecture.

**O-3312**

SOC presenting moderately and poorly differentiated areas (50%) and extended necrotic areas. Few psammoma bodies are observed within the primary ovarian tumor. PDXs derived from this primary serous ovarian cancer recapitulate this morphology either at low or at advanced passages. Large necrotic areas are also present.

**O-1217**

Mixed Müllerian tumors (MMT) or carcinosarcoma. Within the epithelial component, chondrosarcomatous and osteosarcomatous components are observed containing large necrotic areas. Corresponding PDXs bear a similar morphology with serous aspects associated with chondrosarcomatous areas.

**O-1106**

Heterogeneous primary SOC with moderately and poorly differentiated portions containing solid and cribriform structures, important nuclear atypias and elevated mitotic counts. Numerous psammoma bodies are observed with necrotic and inflammatory reaction within the stroma.

PDXs derived from this tumor recapitulate this morphology including psammoma bodies.

**<u>O-3006</u>**

High grade SOC. Note the absence of papillary structures and extended necrotic areas. PDXs reiterate this undifferentiated morphology and large necrotic areas.

**<u>O-1912</u>**

Heterogeneous ovarian carcinoma combining a moderately differentiated SOC and areas of clear-cell carcinoma. Papillary structures are bordered by cancerous cells with numerous mitoses and cellular atypias and necrotic areas. Psammoma bodies are observed.

PDXs derived from this tumor recapitulate this heterogeneity with the presence of both papillary architecture and areas of clear-cell carcinoma.

**<u>O-0822</u>**

Ov-0822 is a primary high-grade serous ovarian carcinoma. Papillae structures are identified with connective-vascular tissues axes. Necrotic areas are rare and Psammoma bodies absent. This tumor is also heterogeneous with the identification of undifferentiated areas with solid aspect and higher cellularity.

PDXs stemming from this tumor maintain this architecture and this heterogeneity. Some of them respect the papillary architecture with various percentages of undifferentiated and solid areas. After the third passage, PDXs examined are undifferentiated with large necrotic areas and high mitotic count.

**<u>O-1458</u>**

High-grade SOC with moderately and poorly differentiated areas. Psammoma bodies are absent and papillae structures coexist with more solid and undifferentiated parts containing large necrotic areas.

PDXs maintain the heterogeneity with identification of rare papillary structures and predominant undifferentiated and solid areas.

**<u>O-1741</u>**

Moderately differentiated SOC with papillary and micro-papillary structures, some rare necrotic areas and psammoma bodies. Portions of undifferentiated are found.

PDXs have a similar morphology with moderately differentiated andpapillary and micro-papillary structures. Necrotic areas, high mitotic count, psammoma bodies are identified.

**<u>Case 2781</u>**

Primary mucinous carcinoma with glandular structures within a connective stroma. Cancerous cells are expanded by the mucus material that is observed also in extracellular position.

Corresponding PDX displays similar morphology with tumoral glands dispersed within a connective stroma. Mucus materials are visualized in intra and extracellular position.

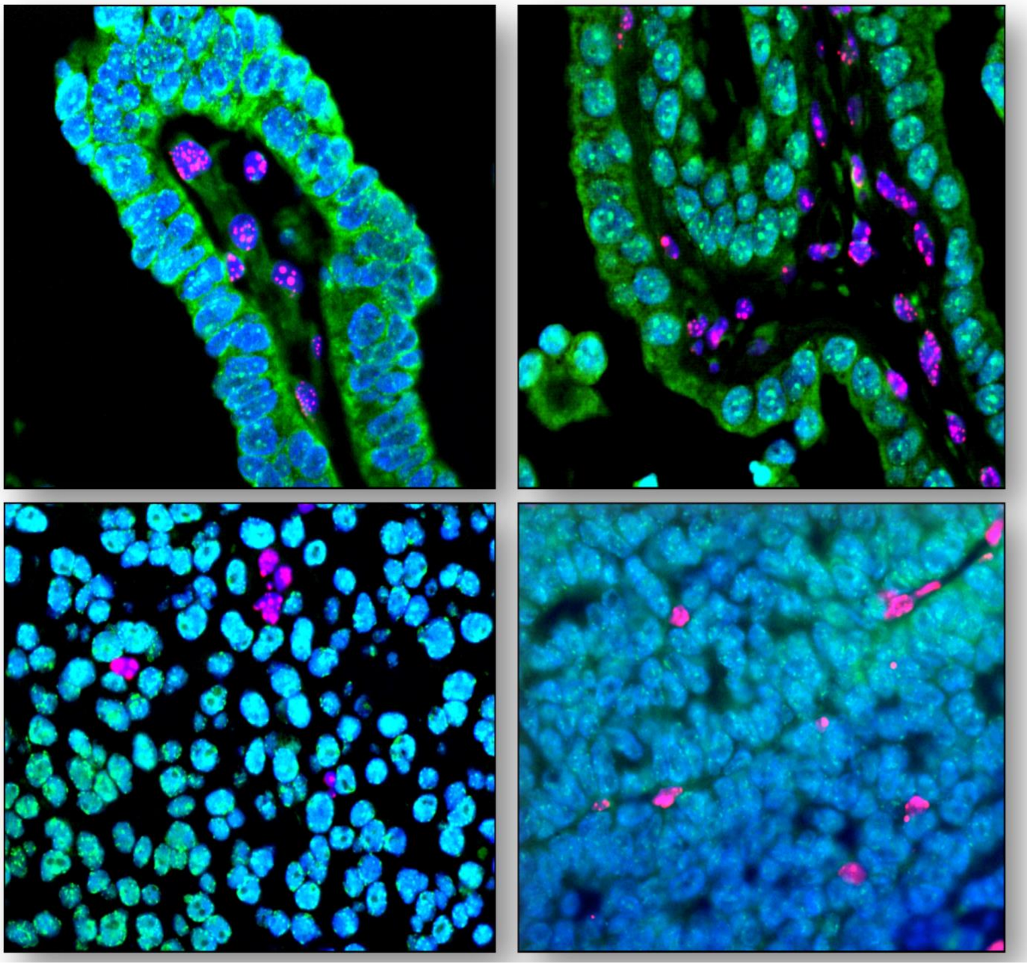

**Supplementary Figure 3**: PDX integrate murine stromal cells. Murine cells were identified by FISH hybridization using red labeled mouse Cot DNA. Human cells were counter labeled with green human Cot DNA.

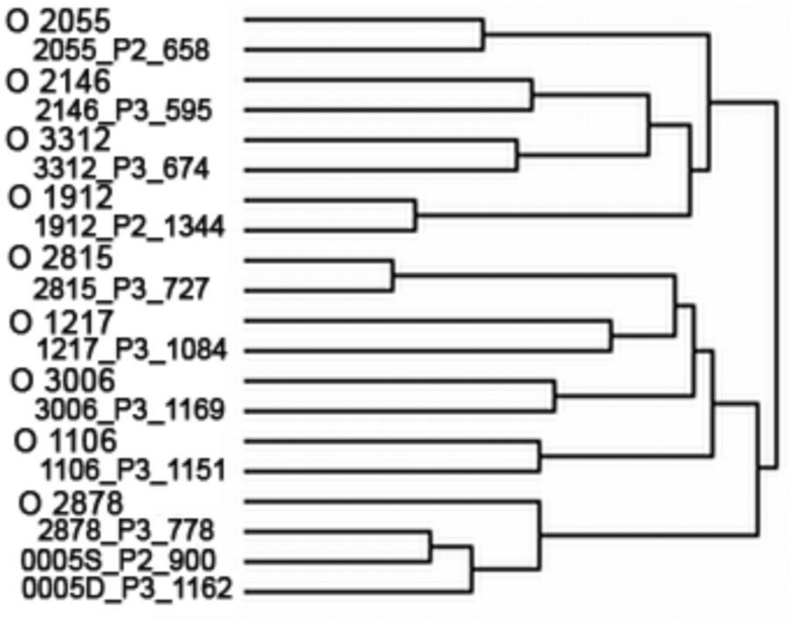


**Supplementary Figure 4**: transcriptomes of tumors of origin and cognate PDXs coclustered indicating conservation of original phenotypic traits.

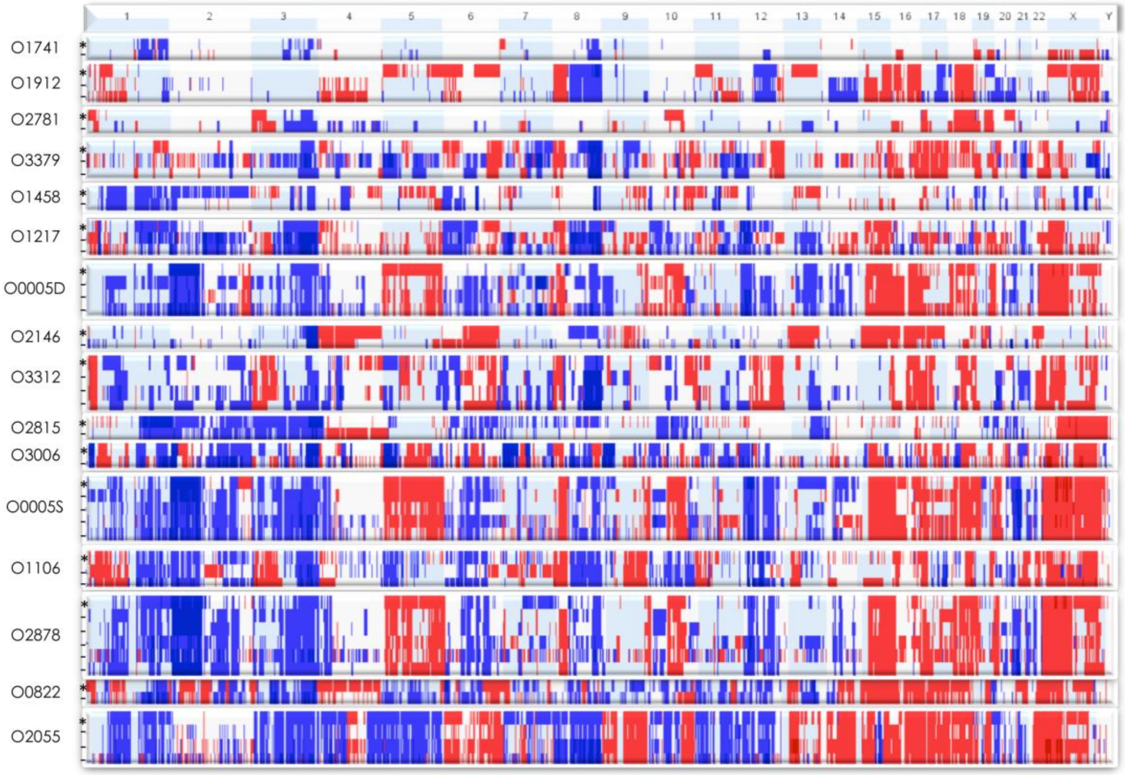


**Supplementary Figure 5** : Whole genome CNC patterns of patient ovarian carcinomas(*) and corresponding PDXs (-). Gains are depicted as blue bars, losses in red; chromosomes are indicated by alternating white and blue columns.

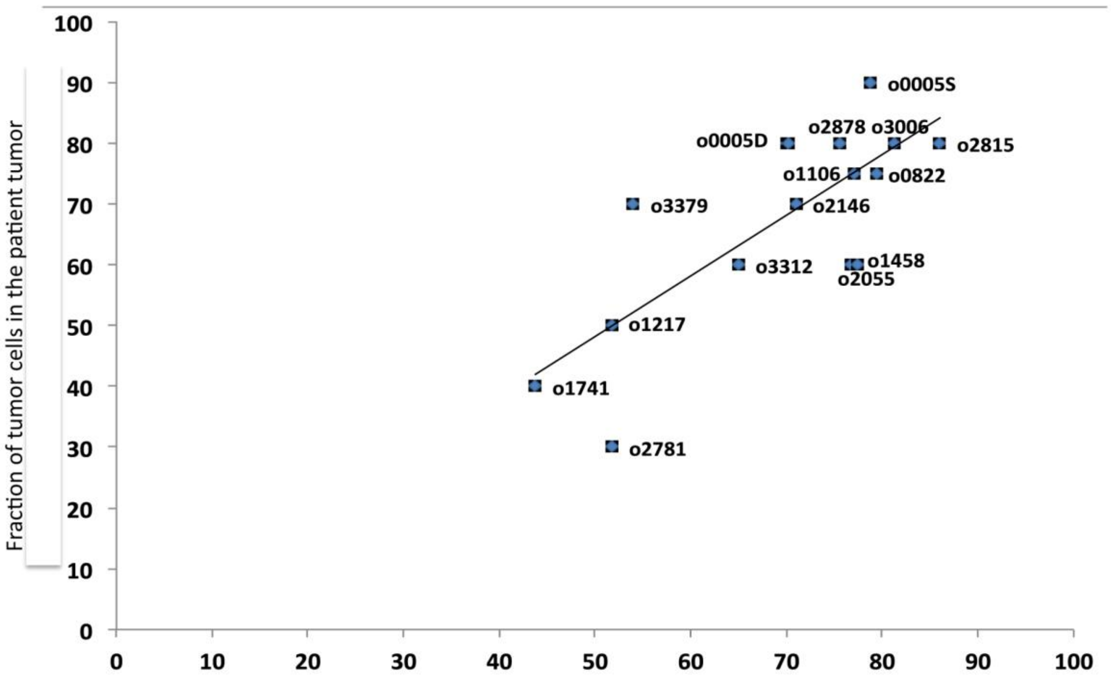


**Supplementary Figure 6**: Pearson correlation coefficient of the CGH profiles of primary tumors and cognate PDX is linked to the proportion of tumor cells in the tumor of origin. As can be seen tumor/PDX pairs that show the lowest Pearson correlation coefficient correspond to the pairs with the lowest proportion of tumor cells in the grafted patient sample.

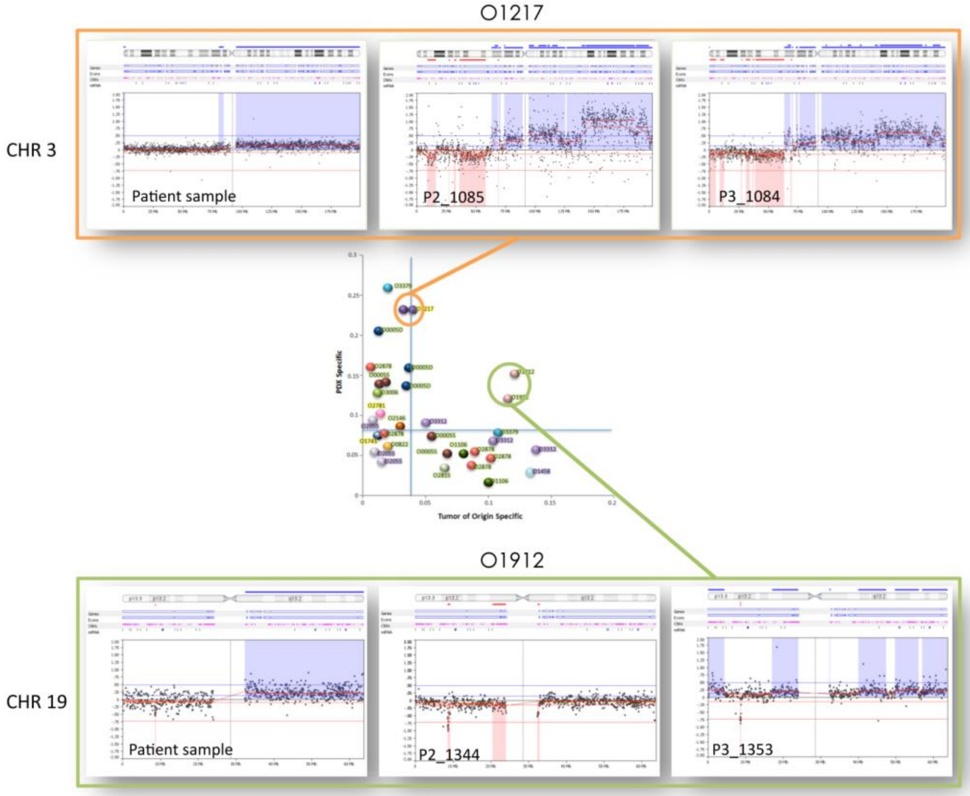


**Supplementary Figure 7**: Examples of CNC fluctuations between patient samples and corresponding PDXs. O1912 P2_1344 illustrates CNCs present in the tumor of origin and lost in the PDX, indicating oligoclonal modifications at this locus. O1217 shows CNCs absent in the tumor of origin and present in the PDXs indicating the unmasking of events in the PDX due to either the oligoclonal nature or the contamination by normal stromal cells in the patient sample. Gains are highlighted in blue areas, losses in red areas.

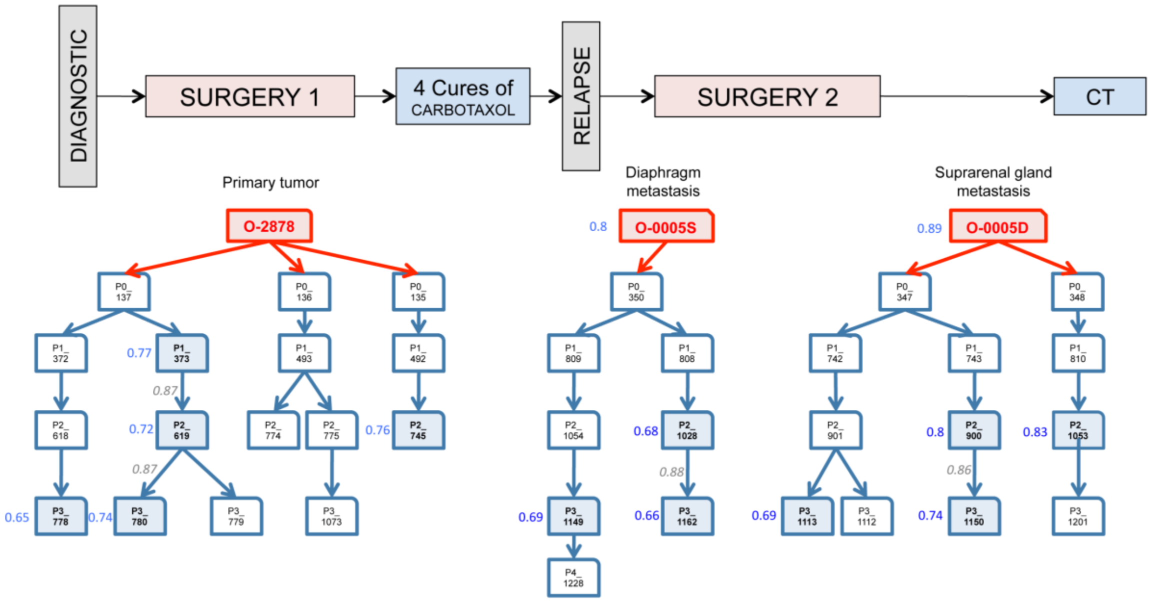


**Supplementary Figure 8**: Case study of one serous ovarian carcinoma : clinical steps and graft tree derived from a primary tumor sample and two asynchronous metastases removed at distinct sites. PDXs whose CNC profiles were studied are colored in light blue. Numbers on the left of each blue box correspond to the Pearson correlation of the CNC profiles of the PDXs with that of the patient sample. Numbers between two blue boxes indicate the Pearson correlation between two consecutive PDXs on the graft tree. Numbers on the left of the red boxes identifying the metastases indicate the correlation between the metastasis and the primary tumor.

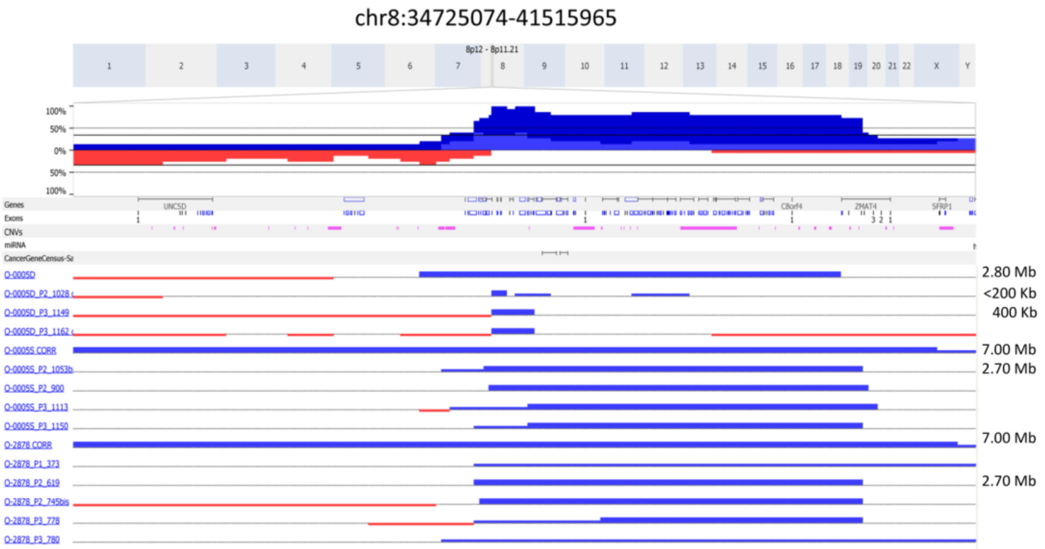


**Supplementary Figure 9:** size of the region amplified at 8p12 in patient samples and corresponding PDXs vary from one sample to another in the O-2878 family . The sizes of the different amplification domains is indicated on the right of the graph. In brief, small amplicons span from 200 to 400 Kb, medium amplicons approximately 3 Mb, large amplicons 7 Mb. Sizes were estimated on the basis of the number of BAC probes showing the copy number increase and from the 5’ end of the telomeric (left) BAC to the 3’ of the centromeric (right) BAC. Actual sizes may be smaller, but the resolution of the array used here would not allow to determine them with greater accuracy.
